# [Retracted] Overexpression of indoleamine 2, 3‑dioxygenase contributes to the repair of human airway epithelial cells inhibited by dexamethasone via affecting the MAPK/ERK signaling pathway

**DOI:** 10.3892/etm.2024.12432

**Published:** 2024-02-19

**Authors:** Shanshan Jia, Pin Guo, Xiangjin Ge, Huanhuan Wu, Junhua Lu, Xiaofang Fan

Exp Ther Med 16:282–290, 2018; DOI: 10.3892/etm.2018.6163

Following the publication of this paper, it was drawn to the Editor’s attention by a concerned reader that certain of the wound-healing assay data shown in Fig. 6B and C on p. 288 were strikingly similar to data that had already been submitted for publication in different form in another article written by different authors at a different research institute [Wang W and Wang J: Toll-like receptor 4 (TLR4)/cyclooxygenase-2 (COX-2) regulates prostate cancer cell proliferation, migration, and invasion by NF-κB activation. Med Sci Monit 24: 5588-5597, 2018].

Owing to the fact that the contentious data in the above article had already been submitted for publication prior to its submission to *Experimental and Therapeutic Medicine,* the Editor has decided that this paper should be retracted from the Journal. The authors were asked for an explanation to account for these concerns, but the Editorial Office did not receive a reply. The Editor apologizes to the readership for any inconvenience caused.

